# 

**Published:** 1897-11-27

**Authors:** 


					the HOSPITAL. ABSOLUTELY PURE, therefore BEST. Nov. 27th, 1897.
CADBURY'S o CADBURY'S
COCOA 1ml /#?T^ COCOA
' The standard of highest purity at present In. /Mk\
attainable."?Lancet. 1??* NO ALKALIES USED.
1?' 583? Vol. xxm. [JttS-a Satueday,
Nov. 27th, 1897.
PRICE TWOPENCE.

				

